# *HOTAIR*: a key regulator in gynecologic cancers

**DOI:** 10.1186/s12935-017-0434-6

**Published:** 2017-06-21

**Authors:** Jing Li, Jing Wang, Yan Zhong, Ruixia Guo, Danxia Chu, Haifeng Qiu, Zhongfu Yuan

**Affiliations:** 1 Department of Oncology, The First Affiliated Hospital of Zhengzhou University, Zhengzhou, 450052 China; 2 Department of Obstetrics and Gynecology, Yantai Yuhuangding Hospital Affiliated to the Medical College of Qingdao University, Yantai, 264000 China; 3 Department of Gynecological Oncology, Linyi Tumor Hospital, Linyi, 276001 China; 4 Department of Obstetrics and Gynecology, The First Affiliated Hospital of Zhengzhou University, No. 1, East Jianshe Road, Erqi District, Zhengzhou, 450052 Henan China

**Keywords:** lncRNAs, *HOTAIR*, Invasion, Metastasis, Cell cycle, Chemoresistance, Radioresistance

## Abstract

Long non-coding RNAs (lncRNAs) play critical roles in the initiation and progression of human cancers. HOX transcript antisense RNA (*HOTAIR*) is an lncRNA localized to the mammalian *HOXC* gene cluster; it can interact with polycomb repressive complex 2 and the lysine-specific histone demethylase/CoREST/REST complex, and it manipulates the expression of various genes. *HOTAIR* promotes tumor invasion and metastasis by silencing tumor suppressors, and activating oncogenes and signaling pathways. *HOTAIR* is deregulated in many human cancers; despite its critical roles in health and disease, the underlying mechanisms governing *HOTAIR* function are unknown. In this review, we summarize the recent findings on the roles of *HOTAIR* in gynecologic cancers.

## Background

Recently, human transcriptome analyses based on deep sequencing and DNA tiling arrays have revealed that only a small fraction of the genome codes for protein, while up to 70% is transcribed [1, 2]. Non-coding transcripts, or non-coding RNAs (ncRNAs), are classified into 2 groups based on their length: small ncRNAs that contain less than 200 nucleotides and long ncRNAs (lncRNAs) that contain more than 200 nucleotides. The latter were originally described in high-throughput sequencing analyses of the full-length mouse genome, and characterized by their lack of open reading frames longer than 100 amino acids [3–6]. Similar to messenger RNAs, lncRNAs are transcribed by RNA polymerase II, then capped at the 5′ end, spliced, and polyadenylated at the 3′ end [7]. The human genome is estimated to contain over 10,000 lncRNAs, which often overlap or are interspersed with coding or non-coding transcripts [2, 8, 9]. Most lncRNAs are evolutionarily conserved and strictly regulated, indicating that they are of functional importance [7, 10]. Many studies have demonstrated that lncRNAs influence the expression of key genes via several mechanisms, including chromatin modification, and transcriptional and post-transcriptional regulation [11].

**Fig. 1:**
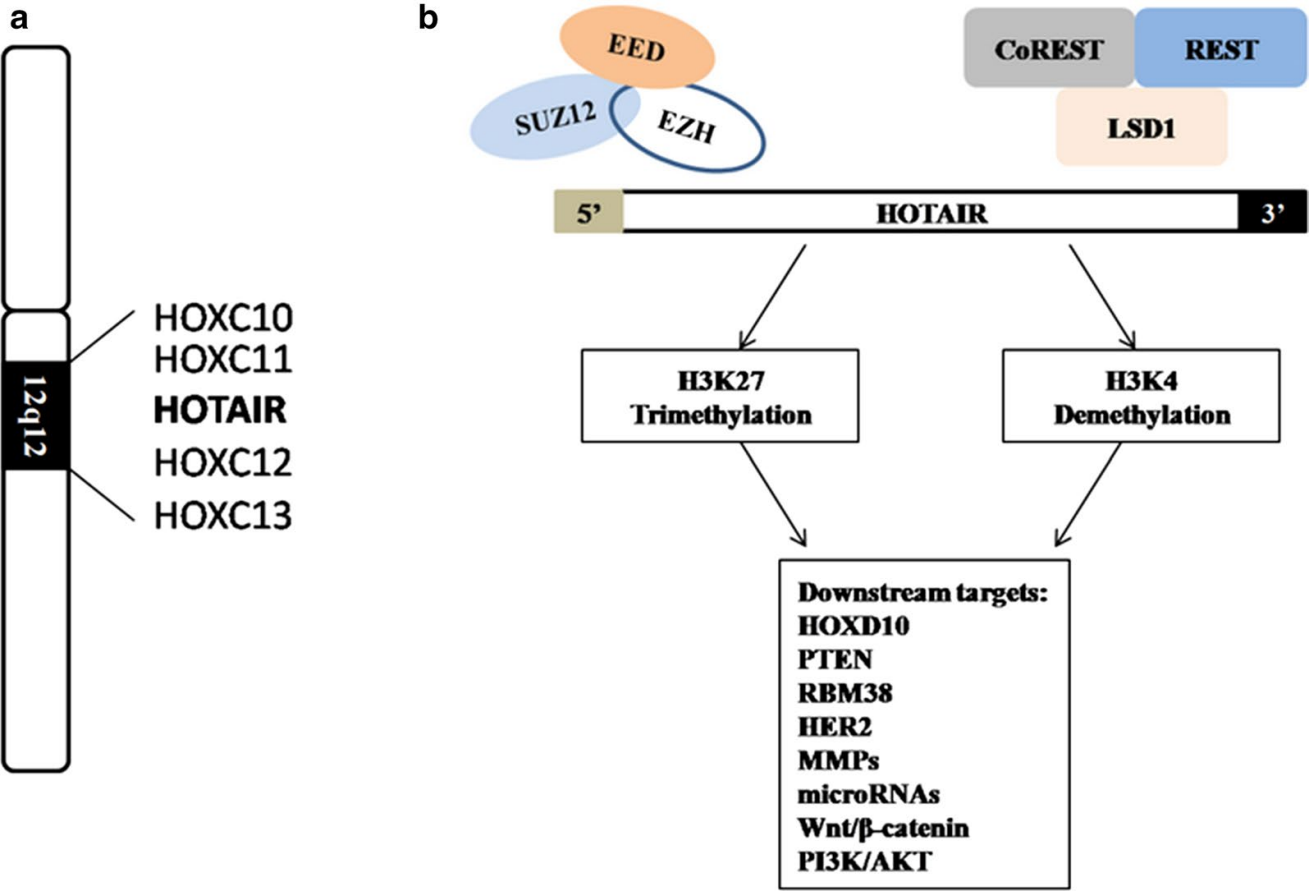
**a** the location of *HOTAIR* in human chromosome 12; **b** the schematic illustration of HOTAIR functions. SUZ12, EED and EZH2 are the three components of PRC complex; CoREST, REST and LSD1 are the three components of LSD1/CoREST/REST complex

HOX transcript antisense RNA (*HOTAIR*) is a well-studied lncRNA, which was first identified by Howard Chang in 2007. This 2.2-kb lncRNA is transcribed from the mammalian *HOXC* gene cluster on chromosome 12q13.13 [12]. As previously reported, *HOTAIR* interacts with the polycomb repressive complex 2 (the PRC2 complex), which consists of the histone H3 lysine 27 (H3K27) methylase EZH2, SUZ12, and EED, at its 5′ end; binds the lysine-specific histone demethylase (LSD1)/CoREST/REST complex via the 3′ domain; and coordinates the targeting of PRC2 and LSD1to chromatin for coupled histone H3K27 methylation and H3 lysine 4 demethylation [13] (Fig. 1).

Given its important roles, it is unsurprising that the deregulation of *HOTAIR* has been implicated in various types of human cancer [14–20]. In breast cancer, enhanced *HOTAIR* expression, which has been detected in both primary tumors and distant metastases, correlates with poor outcomes. *HOTAIR* also increases cancer invasiveness and metastasis by inducing PRC2 retargeting and affecting the methylation of H3 K27 [14]. Moreover, *HOTAIR* is notably elevated in gastric cancer, where it is associated with tumor invasion, metastases, and poor outcomes [16]. While *HOTAIR* promotes cellular invasion and the migration of gastric cancer cells, the downregulation of *HOTAIR* can reverse the epithelial-mesenchymal transition process [21, 22]. In addition, *HOTAIR* can silence key tumor suppressors, like HOXD10, PTEN, and RBM38, and activate key oncogenes and critical signaling pathways, like HER2, Wnt/β-catenin, and PI3K/AKT [19, 23–29]. Intriguingly, abundant *HOTAIR* induced chemoresistance in lung adenocarcinoma; it promoted cell proliferation and cell cycle progression by inhibiting p21 [30].

Recently, we have also found that *HOTAIR* plays a functional role in ovarian, endometrial, and cervical cancers. Herein, we review the functional roles and clinical implications of *HOTAIR* in gynecologic cancers.

## Ovarian cancer

Ovarian cancer is the most fatal gynecologic malignancy worldwide. It is commonly characterized by the development of pelvic and/or abdominal metastases before symptoms present [31]. As previously reported, alterations in *TP53* (the gene for p53) and *BRCA1/2* are the most common genomic events in ovarian cancer; they are associated with increased cancer risk and poor prognosis [32, 33]. Interestingly, single nucleotide polymorphisms in *HOTAIR* were recently found to correlate with susceptibility to ovarian cancer. By genotyping a panel of 1000 Chinese epithelial ovarian cancer patients, Wu et al. demonstrated that rs4759314 and rs7958904 of *HOTAIR* predict the increased susceptibility to epithelial ovarian cancer. For rs4759314, A allele carriers have a higher cancer risk than G allele carriers (OR 1.34) [34]. Consistent with those findings, we determined that rs920778 (T > C) of *HOTAIR* is associated with a statistically significant increase in ovarian cancer risk in 2 separate case–control studies including 329 ovarian cancer patients and 680 cancer-free, age-matched Chinese women. Moreover, our results showed that patients with rs920778 (T > C) obtained a much shorter survival [35]. The above findings evidenced that SNPs of *HOTAIR* might be a potent predictive and prognostic marker for ovarian cancer, which warranted further investigations in large populations and different races.

The deregulation of *HOTAIR* in ovarian cancer has been reported in many studies. By examining the expression of *HOTAIR* in 44 ovarian cancer and 14 normal ovary tissues, Cui et al. found that *HOTAIR* is frequently elevated in ovarian cancer, especially in poorly differentiated cases [36]. Similarly, Qiu et al. detected significant upregulation of *HOTAIR* in ovarian cancer tissues, and this upregulation positively correlated with an advanced International Federation of Gynecology and Obstetrics (FIGO) stage, poor differentiation, and lymph node metastases. Moreover, elevated *HOTAIR* was also an independent prognostic factor for overall survival (OS) and disease-free survival (DFS). The authors also revealed that *HOTAIR* enhanced cellular proliferation, migration, and invasion by upregulating the expression of cyclin E, Bcl-2, caspase-3 and -9, and matrix metalloproteinase (MMP) 9 and MMP3 [26, 37]. In addition, some CD117^+^CD44^+^ ovarian cancer stem cells overexpress *HOTAIR*, and silencing *HOTAIR* with siRNA impaired the migration and invasion of ovarian cancer stem cells [38]. Interestingly, *HOTAIR* could also serve as a competing endogenous RNA to sponge its target microRNAs, thus regulating various cellular behaviors [25, 39]. In ovarian cancer, *HOTAIR* upregulates the expression of *RAB22A* by sponging microRNA-373, thereby enhancing tumor proliferation and invasion, and decreasing apoptosis [40]. Considering its multiple roles, specific targeting and inhibiting *HOTAIR* could be a potent strategy for ovarian cancer treatment in the future.

Although more than 80% of ovarian cancer patients are sensitive to initial platinum-based chemotherapy, most exhibit recurrence and eventually become chemoresistant. Therefore, elucidating the underlying mechanism of chemoresistance is a key issue for ovarian cancer treatment. In carboplatin-treated ovarian cancer patients, Teschendorff et al. found that high levels of *HOTAIR* are associated with poor prognosis [41]. In addition, *HOTAIR* induced resistance to cisplatin in vitro by activating NF-κB, PIK3R3, and MAPK1 [42–45]. These observations strongly suggest that *HOTAIR* plays an important role in inducing chemoresistance in ovarian cancer. In a recent study, we screened the Cancer Genome Atlas (http://cancergenome.nih.gov/) and found that patients with lower *HOTAIR* expression were more sensitive than their counterparts to platinum-based chemotherapy. Furthermore, using in vitro and in vivo assays, we demonstrated that *HOTAIR* promotes proliferation and cell cycle progression, and induces resistance to cisplatin via the activation of Wnt/β-catenin signal; its effects can be neutralized by treatment with XAV-939, an efficacious Wnt/β-catenin inhibitor [46].

Collectively, the pro-cancerous functions of *HOTAIR* have been well demonstrated, and several SNPs can lead to the abnormal upregulation of *HOTAIR*. In addition, targeting *HOTAIR* can overcome the chemoresistance of ovarian cancer, which should be the main topic for us in the future.

## Endometrial cancer

Endometrial cancer mainly comprises endometrioid endometrial cancer (80%), uterine serous papillary cancer, and clear cell cancer, although it also includes rare cancers [47]. During the last few decades, the incidence of endometrial cancer has continually increased, making it one of the most common cancers in women worldwide. Recent studies suggest that overexpression of *HOTAIR* contributes to the initiation and progression of endometrial cancer [48, 49]. Zheng et al. detected overexpression of *HOTAIR* in endometrial cancer tissues compared to healthy, age-matched controls; overexpression was notably associated with the histological grade of the tumor, the presence of lymph node metastases, the depth of myometrial invasion, and invasion of the lymphovascular space. Moreover, higher *HOTAIR* expression predicted poorer OS in those patients [48]. In a subsequent study, it was found that high level of *HOTAIR* correlated with tumor stage, myometrial invasion, and lymph node metastases. Moreover, silencing *HOTAIR* in vitro resulted in extensive G1 phase arrest and sharp declines in cell proliferation, migration, and invasion [49]. Studies of *HOTAIR* in other malignancies have mainly focused on its effects on tumor invasion/migration and the eventual development of metastases; however, *HOTAIR* can strongly enhance cell proliferation by accelerating cell cycle progression. This phenomenon is consistent with the fact that endometrial cancer is mainly localized within the uterine cavity and myometrial invasion occurs in the early stages of disease.

To date, unopposed exposure to estrogen stimulation is the only known etiological factor for endometrial cancer. Interestingly, estradiol is able to induce the expression of *HOTAIR* via direct binding with estrogen response elements, while the estradiol inhibitor genistein downregulates the level of *HOTAIR* in prostate cancer cells [50, 51].

Taken together, the overexpression of *HOTAIR* is a common phenomenon in human endometrial cancer. In vitro assays confirmed its pro-cancerous functions and proposed *HOTAIR* as an effective target for treating endometrial cancer, especially for the anti-estrogen therapy.

## Cervical cancer

Globally, cervical cancer is still the leading malignancy of the female reproductive tract. More than 529,000 patients worldwide, of which more than 80% are in developing countries, are annually diagnosed with cervical cancer [31, 52]. However, the mechanisms underlying the initiation and progression of cervical cancer are still largely unknown. A recent study elucidated a role for *HOTAIR* in the development of cervical cancer by analyzing 218 pairs of cervical cancer and adjacent normal tissues [53]. The study revealed that *HOTAIR* is elevated in cervical cancer tissues, where it correlated with more aggressive biological behaviors, such as late tumor stages, lymph node metastases, and deep cervical invasions. Importantly, high levels of *HOTAIR* were a powerful predictor of poor OS and DFS in these cervical cancer patients [53]. Although they highlight the importance of *HOTAIR* during initiation and progression of cervical cancer, more researches including more patients from different regions are needed in future.

Like that in ovarian cancer, *HOTAIR* polymorphisms have also been found in cervical cancer. In a southern Chinese cervical cancer group, Guo et al. demonstrated that rs920778 is associated with a high cancer risk; the nucleotide change from C to T leads to increased transcriptional activity [54]. Furthermore, we recently demonstrated, in a study of 215 cervical cancer and 430 cancer-free cases, that rs920778 is strongly correlated with the upregulation of *HOTAIR* [55]. Interestingly, we also found that rs920778 could efficiently predict chemo- and radioresistance in cervical cancer patients. Considering the important roles of rs920778, we are now recruiting ovarian and cervical cancer patients for a large cohort study to verify its value as a cancer-risk predictor.

Although cervical cancer usually responds well to surgery and/or radiotherapy, many patients exhibit radioresistance pre- or post-radiotherapy (innate or acquired resistance) and ultimately die of widespread metastasis [56, 57]. In a recent study, we demonstrated that circulating *HOTAIR* is markedly upregulated in the sera of cervical cancer patients, and this upregulation is associated with advanced tumor stage, invasion of the lymphovascular space, and lymph node invasion. Furthermore, a follow-up study demonstrated that high levels of *HOTAIR* positively correlate with tumor relapse and short OS [58]. In another study, we further explored the role of *HOTAIR* in the regulation of the radiosensitivity of cervical cancer. Using immortalized cervical cancer cells and an animal model, we demonstrated that *HOTAIR* reduces radiation-induced apoptosis and leads to cellular radioresistance via targeting p21; in contrast, the knockdown of *HOTAIR* promoted cellular apoptosis and re-sensitized cancer cells to radiotherapy. In addition, we also found that high *HOTAIR* expression predicts cellular radioresistance [59].

Collectively, a large amount of cervical cancer patients presented abnormal *HOTAIR* expression, which predicted high cancer risk, resistance to routine therapies and poor prognosis. Although several factors like SNPs and p21 were involved in, more details were still unrevealed yet.

## Conclusion

In summary, the upregulation of *HOTAIR* frequently occurs in gynecologic malignancies and usually predicts tumor metastases and poor prognosis. In our opinion, three issues should be given priority to be investigated: (1) how to used SNPs of *HOTAIR* for cancer-risk prediction? (2) how to design specific inhibitors of *HOTAIR* for clinical use? (3) to further elucidate how *HOTAIR* participate into chemo- and radio-resistance is urgently required.

## Abbreviations

ncRNA: non-coding RNA
lncRNA: long non-coding RNA
*HOTAIR*: HOX transcript antisense RNA
PRC2: polycomb repressive complex 2
H3K27: histone H3 lysine 27
OS: overall survival
DFS: disease-free survival
MMP: matrix metalloproteinase
